# A Prototype Assay Multiplexing SARS-CoV-2 3CL-Protease and Angiotensin-Converting Enzyme 2 for Saliva-Based Diagnostics in COVID-19

**DOI:** 10.3390/bios13070682

**Published:** 2023-06-27

**Authors:** Vallabh Suresh, Daniel A. Sheik, Tyler C. Detomasi, Tianqi Zhao, Theresa Zepeda, Shyam Saladi, Ummadisetti Chinna Rajesh, Kaleb Byers, Charles S. Craik, Vincent Jo Davisson

**Affiliations:** 1 Department of Medicinal Chemistry and Molecular Pharmacology, Purdue University College of Pharmacy, West Lafayette, IN 47907, USA; sureshv@purdue.edu (V.S.);; 2 Amplified Sciences, Inc., West Lafayette, IN 47906, USA; 3 Department of Pharmaceutical Chemistry, University of California, San Francisco, CA 94143, USAcharles.craik@ucsf.edu (C.S.C.); 4 Neelyx Labs, Wood Dale, IL 60191, USA

**Keywords:** COVID-19, proteases, enzymatic assays

## Abstract

With the current state of COVID-19 changing from a pandemic to being more endemic, the priorities of diagnostics will likely vary from rapid detection to stratification for the treatment of the most vulnerable patients. Such patient stratification can be facilitated using multiple markers, including SARS-CoV-2-specific viral enzymes, like the 3CL protease, and viral-life-cycle-associated host proteins, such as ACE2. To enable future explorations, we have developed a fluorescent and Raman spectroscopic SARS-CoV-2 3CL protease assay that can be run sequentially with a fluorescent ACE2 activity measurement within the same sample. Our prototype assay functions well in saliva, enabling non-invasive sampling. ACE2 and 3CL protease activity can be run with minimal sample volumes in 30 min. To test the prototype, a small initial cohort of eight clinical samples was used to check if the assay could differentiate COVID-19-positive and -negative samples. Though these small clinical cohort samples did not reach statistical significance, results trended as expected. The high sensitivity of the assay also allowed the detection of a low-activity 3CL protease mutant.

## 1. Introduction

The coronavirus disease 2019 (COVID-19) pandemic was the largest global health emergency in the past century. In 2022, total confirmed cases reached over half a billion worldwide, of which nearly 7 million deaths have been recorded [1]. These numbers of cases are likely to be skewed because of incomplete reporting. Estimations based on population statistics and excess mortality indicate more than 18 million deaths [2]. The true magnitude of the loss of life during the pandemic is likely much greater [3]. Diagnostics will continue to play an important role in the management of such large pandemic and endemic disease scenarios [4].

The advantages of each diagnostic modality present unique opportunities for clinical decision-making. Nucleic acid tests, most often detecting amplified viral RNA, are highly sensitive and can discriminate virus-positive and virus-negative patients in less than 20 minutes using isothermal amplification methods [5]. During the COVID-19 pandemic, testing aimed to limit the spread of the disease. While sometimes generating false positive results because of high cycle numbers, these nucleic acid tests completely avoided false negative results and were thus most useful for limiting the spread of the virus [6,7].

As COVID-19 becomes endemic in current times, the role of diagnostics redirects to (1) correctly understanding vaccine response and the prior epidemiology of the disease and (2) decisions for patients’ treatment options [3,8,9,10]. These applications require avoidance of both false positive and false negative results in order to (1) accurately understand infection rates and protection because of immunity and (2) prevent unneeded treatment of non-infected patients [11,12,13]. Serological tests detect antibodies produced against the SARS-CoV-2 nucleocapsid protein and 3CL protease [14]. The United States Centers for Disease Control (CDC) has adopted a serological surveillance program aimed at understanding the previous infection in patients. These studies will provide a basis for assessing true infection and re-infection rates, informing successes in protective immunity after infection or vaccination.

Enzyme assays offer an alternative approach to potentially stratify patients most in need of treatment [15,16]. Measurement of angiotensin-converting enzyme 2 (ACE2) activities in saliva have been related to the natural resistance of patients against COVID-19 symptoms [17]. A corollary is that ACE2 activity shows potential use to assess disease severity in infected patients [18]. Nevertheless, ACE2 levels fluctuate for many reasons not related to COVID-19 [19,20,21]. A hypothesis under consideration is that pairing severity- or resistance-associated enzyme activity measurements, such as ACE2, with virus-specific biomarkers, such as 3CL protease activity, could inform medical practice. Based on the life cycle of the virus, the presence of active 3CL protease is associated with an ongoing and active infection [22]. This is a significant differentiating feature of enzyme assays from serological testing, which can read positive because of the long tenure of antibodies in the body because of past infections. By combining multiple enzyme assays, we suspect it is possible to (1) assess whether a patient is actively infected, (2) determine how severe symptoms will be as the infection progresses, and (3) direct treatment to the most at-risk patients. 

Various assays have been developed to measure the activity of the SARS-CoV-2 3CL protease in patient samples, though none are specifically developed for multiplexing with other enzymes such as ACE2 [23,24,25,26]. However, the majority of 3CL protease assays have been designed for inhibitor discovery and not clinical samples of bodily fluids [27,28,29,30,31]. 

To enable an investigation of the value of enzyme assays in COVID-19 diagnosis, a first iteration of a novel 3CL protease activity assay in combination with an ACE2 assay has been developed [18]. This duplex assay is designed to run on saliva samples, though it will likely tolerate a variety of other biofluids [32]. A magnetic bead platform with a novel labeled peptide substrate, capable of both fluorescence and surface-enhanced resonance Raman spectroscopy (SERS) readouts, is reported [33,34,35]. This new 3CL protease assay can be run on the same sample immediately after ACE2 assay with readouts of both enzymes in 30 min using minimal sample volumes at concentrations below the dimer dissociation constant. The sensitivity of the assay is demonstrated here using a 3CL protease mutant with an impaired dimerization interface. 

## 2. Materials and Methods

### 2.1. Materials

Synthetic procedures for the dimeric rhodamine 6G dye used in the present study have been reported previously [34,35]. This dye was coupled to the N-terminus of resin-bound biotinylated peptides, prepared using a liberty blue synthesizer (CEM, Matthews, NC, USA). All Fmoc-amino acids, including Fmoc-Lys(Biotin)-OH and ethyl-(hydroxyamino)-cyanoacetate (Oxyma, 26426), were purchased from Chem Impex. Di-isopropyl carbodiimide (DIC, AC446181000), 2-(6-Chloro-1H-benzotriazole-1-yl)-1,1,3,3-tetramethylaminium hexafluorophosphate (HCTU, NC0576737), dichloromethane (DCM, MK-4879-4), trifluoroacetic acid (TFA, AC139720025), zinc chloride (ZnCl_2_, 208086), sodium ethylenediamine tetraacetate (EDTA, E5391), hydrochloric acid (HCl, A142-212), dithiothreitol (DTT, D9760), diethyl ether (AC12399-0050), acetonitrile for HPLC (A998), and water for HPLC (600-30-13) were all purchased from Fisher Scientific (Pittsburgh, PA, USA). *N*,*N*′-diisopropylethylamine (DIEA, D125806), *N*,*N*′-dimethylformamide (DMF, 319937), tris hydrochloride (10812846801), and sodium chloride (NaCl, S9625-1KG) were purchased from Sigma Aldrich (St. Louis, MO, USA). Triisopropylsilane (TIPS, A187865) was purchased from Ambeed (Arlington Heights, IL, USA). Tween^®^ 20 (97062-332) was purchased from VWR (Radnor, PA, USA). Recombinant SARS-CoV-2 3CL protease (E-720), recombinant angiotensin-converting enzyme 2 (933-ZN-10), and Mca-YVADAPK(Dnp)-OH fluorogenic peptide substrate (ES007) were purchased from R&D Systems (Minneapolis, MN, USA). Artificial saliva was purchased from Biochemazone (Edmonton, AB, Canada). Pooled human saliva (IRHUSL5ML) was purchased from Innovative Research. Streptavidin-blocked sera-mag speed beads (21152104011150) were manufactured by Cytiva (Marlborough, MA, USA). Pepstatin A (S7381) was purchased from Selleckchem (Houston, TX, USA). Lysozyme (Aldrich, L6876), trypsin (Research Products International, Mount Prospect, IL, USA, cat. no. T70010-1.0), soybean trypsin inhibitor (Aldrich, T6522), pepsin (R&D Systems, 6186-AS-010), and bovine serum albumin (Sigma Aldrich, A7906-50G) were purchased from their manufacturers. In addition, 50 nm citrate-capped silver nanoparticles (1 mg/mL, AGCB50-5M) were purchased from nanoComposix (San Diego, CA, USA). A 384-shallow-well, black, flat bottom plate (Thomas Scientific, Swedesboro, NJ, USA, cat. no. 1230M76) and 0.5 mL sterile centrifuge tubes (MTC Bio, Sayreville, NJ, USA, cat. no. C2007) were used. Alpha-cyano-4-hydroxy-cinnamic acid (145505-5G) was purchased from Sigma Aldrich. All assay samples were heated on an incubator (VWR, 75838-270) equipped with a 30-tube block (VWR, 13259-000) and a temperature control probe (VWR, 11301-112). All fluorescence readings were measured on a Biotek Synergy H1 Plate Reader. SERS spectra were obtained using a probe-based Raman spectrometer (Wasatch Photonics Inc., Logan, UT, USA model WP-532-SR-IC) with a 532 nm laser source, 50 mW laser power, 25 µm slit width, and standard lenses at a 11 mm working distance using 20–30 ms integration time. Procedures for peptide synthesis and protein production are given in the supplementary information.

### 2.2. Preparation of Assay Buffers I and II

For the first screening with additives, an initial Buffer I was prepared to contain 20 mM Tris, 150 mM NaCl, and 0.05 *v*/*v*% Tween 20. The pH was adjusted to 7.4 using HCl. After screening, 1 mM EDTA and 1 mg/mL soybean trypsin inhibitor were added freshly to Buffer I prior to each experiment to produce a completed Buffer II. Buffer II was used or mixed with artificial or pooled human saliva when mimicking patient samples.

### 2.3. Preparation of Protease Standards

Recombinant 3CL protease versions were prepared using modified procedures described in Supporting Information [36,37]. The purified proteases, ACE2, and other proteins were diluted with Buffer II or saliva/Buffer II mixtures to produce 2× protease standards. When needed, these 2× samples were frozen and thawed or otherwise processed prior to activity assays.

### 2.4. Preparation of Magnetic Beads

Streptavidin-coated magnetic beads were loaded with 3CL substrate in bulk. Generally, 0.05 mL aliquots of bead stock (10 mg/mL) were twice washed with water and separated via magnetic separation with a magnetic rack. The washed beads were then resuspended in and incubated with a 0.1 mL volume of 0.05 mM 3CL substrate at room temperature for 20 min. After incubation, the beads were washed 6 times with Buffer I using double the original bead aliquot volume and again isolated via magnetic separation. After the final wash, loaded beads were resuspended in Buffer I at double the original bead aliquot volume.

### 2.5. Preparation of ACE2 Fluorescent Substrate

ACE2 substrate peptide, as a DMSO stock (2 mM), was diluted to a 2× working concentration of 40 µM in Buffer I with 20 µM ZnCl_2_. This stock was used in ACE2 and duplex assays.

### 2.6. 3CL Protease Assay

Equal volumes of samples containing 3CL protease and loaded magnetic bead stocks were added into PCR tubes. These samples were heated in an incubator at 37 °C for either 3 h (to establish standard curves) or 20 min (duplex protocol). The beads were then sedimented using a magnetic rack, and liquid supernatants (8 µL each) were pipetted into a black 384-well shallow plate. A Biotek H1 plate reader was used to analyze fluorescence (Ex: 516 nm; Em: 557 nm; gain: 80). 

### 2.7. ACE2 Assay

Equal volumes of ACE2-containing samples and fluorescent substrate solution were added into wells of 384-well black plates to initiate reactions. The reactions were carried out at room temperature. For the kinetic assays, solution fluorescence (Ex: 320 nm; Em: 405 nm; gain: 80) was read every 20 s for 5 min. Fluorescence endpoints at 10 min were recorded for all other experiments.

### 2.8. 30-Minute Sequential ACE2-3CL Assay

For saliva samples containing both ACE2 and 3CL protease, the ACE2 reaction was carried out using 10 µL of sample and 10 µL of fluorescent ACE2 substrate solution. After collecting fluorescent endpoints after 10 min, 5 µL of the reaction solution was mixed with 5 µL of Buffer II, containing 2 mM EDTA and 2 mg/mL soybean trypsin inhibitor, to quench any other proteases aside from 3CL protease. Next, 5 µL of this newly quenched sample was mixed with 5 µL of loaded magnetic bead reagent. This reaction mixture was incubated at 37 °C for 20 min. The supernatant was collected, and fluorescence was measured as described above.

### 2.9. Raman Spectroscopy

One microliter of sample was diluted into 99 µL of 50 mM acetate buffer at pH 4.5 supplemented with 100 mM NaCl and 0.1% Tween to promote aggregation and SERS enhancement; 5 µL of the newly diluted sample or a sample containing only acetate buffer was placed into a well of 384-shallow-well plates prior to addition of 10 µL of a 1 mg/mL suspension of 50 nm Ag nanoparticles [38]. The suspension was mixed by pipetting the sample up and down and then was allowed to approach equilibrium for 5 min. After this time, the sample was subjected to Raman spectroscopy to obtain the SERS spectra. The sample containing only buffer was used for background subtraction by the Enlighten software package used to obtain spectra. Spectral data were processed for peak areas using the OPUS 8.2.28 software package. Graphs of the data were prepared using GraphPad Prism, and the error bars represent the standard deviation from replicate measurements.

### 2.10. Matrix-Assisted Laser Desorption/Ionization Time-of-Flight Mass Spectrometry (MALDI-TOF MS)

All data collection was performed using a Voyager DE mass spectrometer. Alpha-cyano-4-hydroxycinnamic acid (CHCA), as a saturated solution in 1:1 acetonitrile:water with 0.1% trifluoroacetic acid, was used as the matrix. Samples taken from 3CL protease wild-type and mutant enzymatic reactions were prepared using a modified three-layer method [39]. Briefly, 1 µL of matrix was allowed to dry at marked spots on a stainless-steel plate. On top of the dried matrix spot, 0.5 µL of analyte solution was placed and air dried further. Finally, a second 1 µL droplet of matrix was added and air dried. The resulting spots were analyzed using the mass spectrometer.

### 2.11. Collection of Clinical Samples (IRB, etc.)

Residual saliva specimens were collected from patients presenting for diagnostic COVID-19 RT-PCR testing. After adequate samples had been aliquoted for routine care, they were de-identified and frozen at −80 °C until the time of testing. Informed consent was obtained from all subjects. The study was conducted in accordance with the Declaration of Helsinki and approved by the Heartland Institutional Review Board (HIRB Project No. 092222-416, approved 22 September 2022). 

## 3. Results

**Scheme 1:**
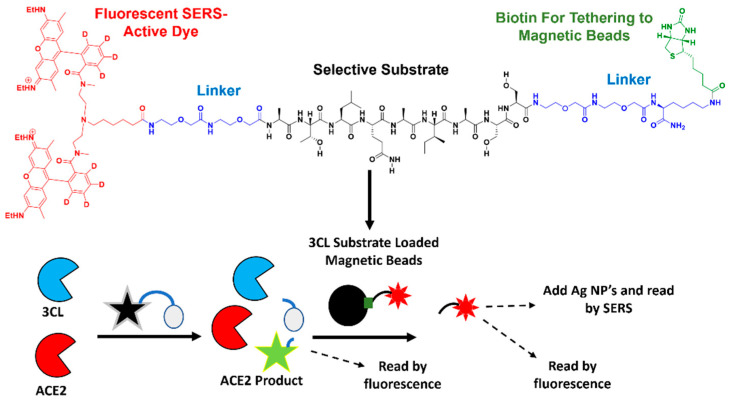
Design of a selective substrate and its usage in a sequential dual-protease assay allowing measurement of ACE2 and 3CL protease activity in the same sample.

In this work, a salivary SARS-CoV-2 3CL protease assay is demonstrated, which can be run sequentially after an ACE2 assay, without any sensor-reagent interferences in either assay. The assay design is described in Scheme 1. The peptide substrate is adapted from a previously identified 3CL protease cleavage sequence before NSP7 in the polyprotein (ATLQAIAS), an N-terminal dimeric dye to enable assay readout, and a C-terminal biotinylated lysine for immobilization to magnetic beads [33,36]. The lysine-biotin residue is separated from the rest of the peptide by serine-diethylene glycol spacer to preserve the biotin-streptavidin complex [35,40]. After immobilization on streptavidin-conjugated blocked magnetic beads, the unbound substrate is washed away using an excess Buffer I. The suspension of loaded beads is mixed in equal volumes with Buffer II or saliva samples containing 3CL protease. Protease cleavage of the substrate liberates a dye-tagged product into solution, which can be measured using fluorescence or Raman instrumentation. An advantage of this assay format is that y-axes can be easily normalized to product concentrations using a standard curve of dye product. This normalization allows for a readout in micromoles of product, which directly relates to enzyme turnover as opposed to arbitrary fluorescence, which indirectly correlates.

**Figure 1:**
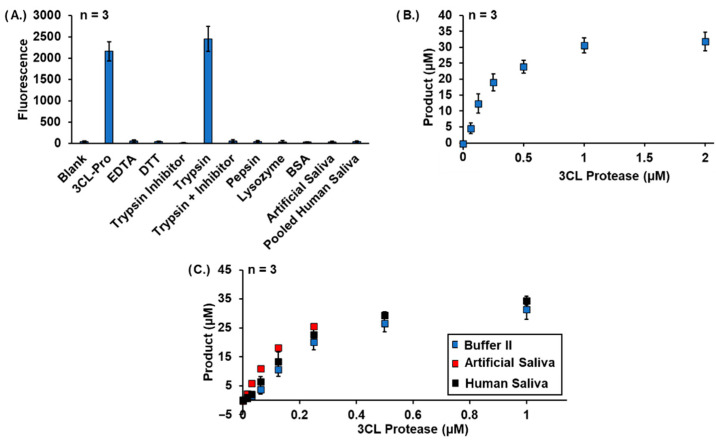
(**A**). Selectivity of the assay for a panel of additives; soybean trypsin inhibitor used at 1 mg/mL; all other proteins were used at 1 µM; EDTA and DTT were used at 1 mM; saliva matrices were used at 1:3 ratio with Buffer I; (**B**). 3CL protease concentration dependence of the assay; Y−axes normalized to concentration of product generated in the reaction using a pre-generated standard curve of the substrate dye (Figure S2); (**C**). fluorescent assays showing enzyme concentration dependence under final conditions in Buffer II (150 mM NaCl, 1 mM EDTA, 1 mg/mL soybean trypsin inhibitor), or 1:3 mixtures of saliva matrices and Buffer II.

To ensure selectivity under assay conditions, the peptide substrate was exposed to a variety of additives: a metal-chelator, a reducing agent, proteases, common proteins, and relevant matrices—artificial and pooled human saliva (Figure 1A). The substrate showed an enzyme-concentration-dependent signal generation with recombinant SARS-CoV-2 3CL protease as expected (Figure 1B). However, a trypsin-catalyzed reaction was also observed. While the assay did not generate any signal in saliva matrices (in the absence of 3CL protease) in our initial tests, trypsin-like proteases do exist in saliva and present the possibility of false positives in larger cohorts of saliva samples [41]. To circumvent any potential false positives, soybean trypsin inhibitor was used as an additive to completely inhibit trypsin in the saliva-based assay, consistent with a previous report [42]. Consistent with a previous report, we found that a reducing agent was not required to get activity in our assay [28]. 

Based on the initial screen of compatible additives, standard assay conditions were developed using fully modeled matrix effects that can occur in clinical samples by incorporating ethylenediaminetetraacetate (EDTA) and soybean trypsin inhibitor. Because metal ions are known to inhibit 3CL protease, the addition of EDTA was essential to prevent loss of activity and variation between samples [43]. Using these conditions, fluorescence assay responses were nearly identical regardless of whether 3CL protease was formulated in Buffer II, artificial saliva, or pooled human saliva (Figure 1C). Using standard curves of dye-peptide conjugates prepared separately (Figure S2), the assays can be normalized for product production. By calculating three standard deviations above the signals generated in samples lacking the enzyme, the limits of detection are between 16 nM and 32 nM of 3CL protease. Using nine standard deviations above the samples lacking enzyme, the limit of quantitation is between 32 nM and 64 nM enzyme, and the assay is linear up to 500 nM. The lack of difference between artificial and pooled saliva samples indicates that this assay is fully robust against other enzymes found in human saliva.

**Figure 2:**
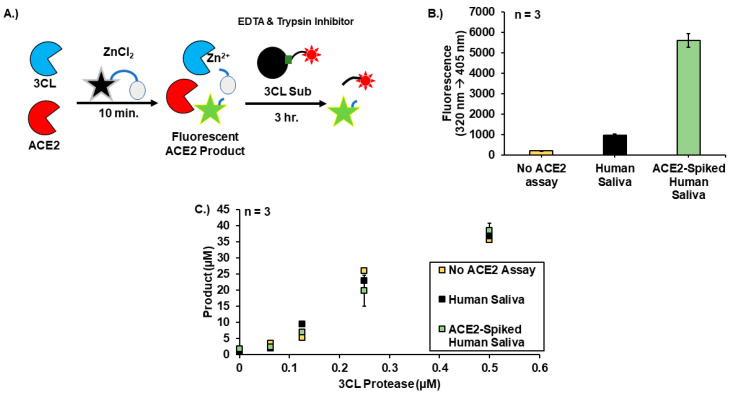
(**A**). Assay schematic in which ACE2 activity is first measured followed by 3CL protease immediately after in the same sample; (**B**). fluorescence background generated by ACE2 reaction in different matrices; (**C**). 3CL protease fluorescence readout standardized for product formation in the presence of the background generated in the ACE2 assay prior.

A procedure was developed for the measurement of ACE2 and 3CL protease in the same sample. ACE2 assays using a quenched substrate for fluorescence-resonance energy transfer (FRET) analysis were obtained with saliva samples spiked with both enzymes. These assays are run with the addition of zinc salts to support enzymatic activity by ACE2. Using the fluorescent solutions generated by the ACE2 assay, 3CL protease assays were run with the addition of EDTA, trypsin inhibitor, and substrate-loaded magnetic beads. The successful observation of 3CL protease activity indicates that the catalytic activity can be recovered after treatment with enzyme-inhibiting zinc (Figure 2) [43,44]. The comparison of assays run in ACE2-spiked and non-spiked human saliva also shows that background interference from fluorescent ACE2 products did not significantly affect the assay responses through the entire linear range of 3CL protease previously determined.

**Figure 3:**
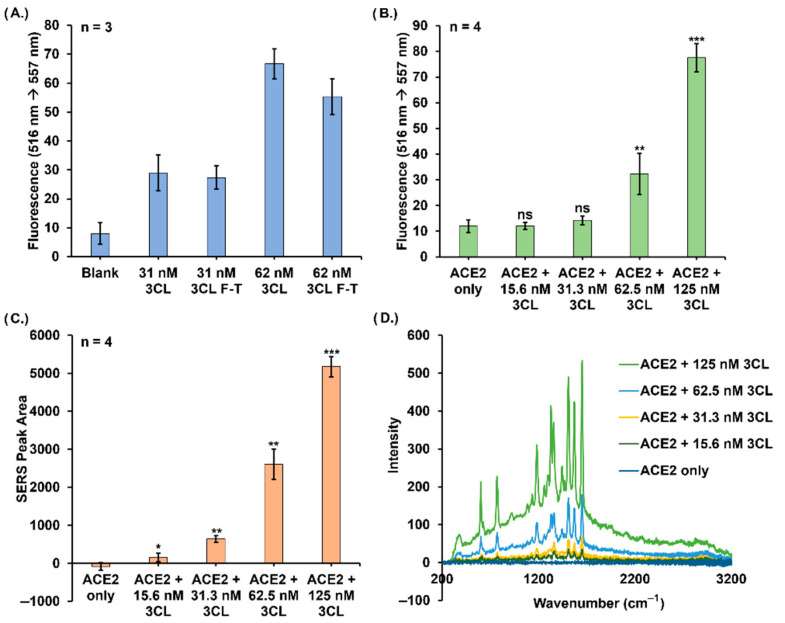
(**A**). Twenty-minute 3CL protease assays differentiate blank and low−concentration samples even after a pre−measurement freeze–thaw cycle; (**B**). at 62 nM or above, 3CL protease samples can be differentiated by fluorescence from blank samples after ACE2 measurement; (**C**). SERS allows more sensitive detection down to 15.6 nM 3CL protease, even after ACE2 reaction; (**D**). SERS spectra associated with these samples. * *p* < 0.05, ** *p* < 0.01, *** *p* < 0.001 according to two-tailed paired heteroscedastic *t*-tests between samples containing 3CL protease and ones that do not.

A more time-efficient 3CL protease assay procedure was next demonstrated to allow for practical use in clinical diagnostics. Clinically relevant assays could benefit from rapid turnaround times when simple confirmations of 3CL-protease samples are sufficient. Using 20-min reaction times, assays clearly differentiated 3CL-spiked and non-spiked saliva samples (Figure 3). Importantly, enzyme activity was still detected after one freeze–thaw cycle. This feature enables the analysis of samples shipped frozen to central diagnostic laboratories. Even in the background of fluorescent product generated in the ACE2 assay, this 20-min procedure was still performed as intended. The fluorescence, dual-enzyme assay could not distinguish between 3CL-spiked and non-spiked samples at the two lowest tested 3CL protease concentrations (15.6 nM and 31.3 nM). This limitation is perhaps due to the fluorescence background signal arising from the dye product generated in the ACE2 assay. Nevertheless, SERS detection distinguished these samples, indicative of the expected advantages of this ultrasensitive technique. These studies prototype a clinically relevant 30-minute duplex assay for measuring both enzymes in the same sample. The limit of detection in the time-efficient fluorescence assay is approximately 62 nM of 3CL protease and is a factor of 2–4 lower when SERS is utilized for detection.

Utilizing the time-efficient highly sensitive SARS-CoV-2 3CLPro assay protocol, clinical saliva samples were evaluated without a pre-analytical concentration step. The sample cohort included four COVID-19-positive (PCR-confirmed) and four negative patients, and each was assayed for ACE2 and 3CL protease (Figures S3–S5). While these data trended toward a difference between the two groups, it did not reach statistical significance. Samples from the COVID-19 positive tended very weakly toward a higher fluorescence value in both the ACE2 assay and 3CL protease assays. Interestingly, the ratio of fluorescence between 3CL protease and ACE2 assays showed a larger difference between COVID-19-positive and -negative samples, though this also did not reach significance (Figure S5). When SERS was utilized, no signal was detected in any samples. These results are consistent with the concentrations of SARS-CoV-2 3CLPro in human saliva not reaching the levels needed for active dimers. Based upon the limit of detection for this assay, it is anticipated that the enzyme would be <30 nM in a human saliva concentration. 

Prior reports attempting to detect 3CL protease in saliva suggest a need for a pre-analytical step to concentrate protein. SARS-CoV-2 3CL protease has been shown to be catalytically active as a dimer with a dissociation constant in the range of 1.0–2.5 µM [45,46,47]. In saliva, the concentration of the protease is likely lower than the dimerization dissociation constant. Differentiation of COVID-19-positive and -negative samples was not directly demonstrated using other 3CL protease assays [23,24,25]. However, one recent study did show a clear differentiation between COVID-19-positive and -negative patients using an antibody-functionalized carbon paper electrode and an associated cyclic voltammetry assay [26]. By concentrating the protease on a test strip surface, the protease would be able to form a catalytically active dimer that can more easily be detected using an electrochemical method. Antibody-functionalized carbon paper was used to capture as low as 1 µg/mL (~30 nM) 3CL-protease, and the resulting clinical assay could detect as low as 6.6 µg/mL (~200 nM). While these concentrations are much higher than our own assay’s detection limits in Figure 3 (~15 nM), our failure to discriminate COVID-19-positive and -negative clinical samples suggests that the success of the electrochemical method is due to the protein concentration step. 

**Figure 4:**
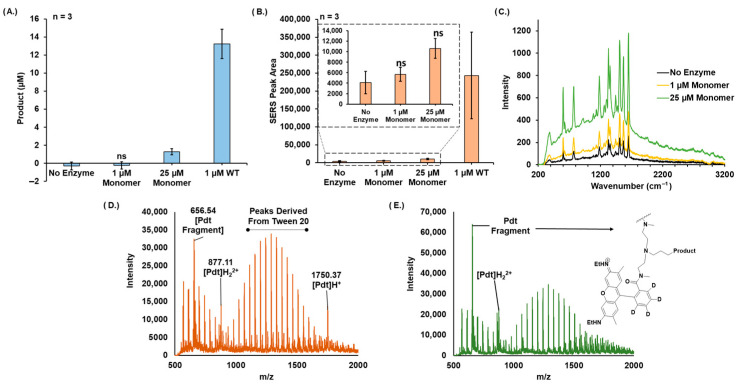
(**A**). Fluorescent assays show the potential activity of a mutant enzyme (termed monomer here) with a disrupted dimer interface. (**B**). Quantified SERS activity for the monomer and WT enzyme. SERS further identified similar enzyme activity at a lower concentration of the mutant enzyme. (**C**). SERS spectra are generated by reactions of the monomer enzyme. (**D**). MALDI-TOF MS spectra of a reaction of 1 µM wild-type 3CL protease. (**E**). MALDI-TOF MS spectra of a reaction of 25 µM mutant monomer 3CL protease. Statistical significance was determined using two−tailed paired heteroscedastic *t*-tests between samples containing enzymes and ones that do not.

Since this assay demonstrated an ability to detect catalytic activity at protein concentrations far below the dimer dissociation constant, a SARS-CoV-2 3CL protease mutant was selected for the study of the catalytic activity. This mutant protein contains an extra serine and asparagine residue at the N-terminus that interferes with the ability of 3CL protease to dimerize. The protein is primarily in the monomeric form as determined by SEC during purification (procedures for protein production are provided in the supplementary information). Unsurprisingly, this mutant did not exhibit observable activity in the fluorescence-based assay, except at very high enzyme concentrations (25 µM) (Figure 4A). In the SERS assay, the mutant enzyme did not present a signal that was significantly higher than the background in these conditions; however, a slight concentration dependence signal was observed (Figure 4B,C). To confirm that this signal was a product generated by protease cleavage, we utilized MALDI-TOF MS (Figure 4D,E). Analysis of the wild type 3CL protease reaction mixtures showed peaks associated with the singly charged dye-labeled proteolysis product (Pdt-H^+^), a doubly charged product (PdtH_2_^2+^), and a doubly charged fragment arising from the product. Both doubly charged peaks were also observed in the 25 µM mutant reaction mixture, though they could not be detected in the 1 µM mutant reaction mixture likely because of a lack of sensitivity of the mass spectrometry method in these complex samples. 

Taken together, these data suggest that the SERS signals detected are very likely due to proteolysis catalyzed by the mutant enzyme and are not nonspecific signals. Based on the calculated product concentrations, the wild-type enzyme generated >12 turnovers, while the mutant, at both 1 and 25 µM, generated ~0.06 turnovers. The observed activity of the mutant is thus less than 1% of the wild-type enzyme. We hypothesize that the reactions catalyzed by the mutant are due to a small concentration of catalytically active dimer, formed only in highly concentrated solutions. To our knowledge, this is the first time an assay has had suitable sensitivity to quantify this low number of turnover events for 3CL protease in a format that can multiplex enzyme activities.

## 4. Discussion

Based on what is known about virus life cycle, 3CL protease assays have the potential to distinguish actively and acutely infected COVID-19 patients from those whose infection has passed [22]. The ACE2 activity biomarker has previously shown potential in stratifying patients who are severely ill and distinguishing potentially infection-resistant individuals [17,18]. The combination of these two approaches could provide an effective diagnostic tool to direct COVID-19 treatments to patients who need them most. 

In this work, we have demonstrated sequential duplex assays of both ACE2 and 3CL protease. These assays use a combination of designed 3CL protease substrate and optimized assay additives to enable robustness against false positives. The entire procedure can be conducted in 30 min. This work also demonstrated that SERS measurement can be more sensitive near limits of detection. Though extremely powerful for detecting low concentrations of analytes, SERS is an underappreciated technique for bioanalysis. Our demonstration of SERS’s robustness to a fluorescent background is expected to promote its usage in clinical diagnostics. 

Our assays detected signal from a mutant enzyme was shown to have less than 1% of the activity of the wild type. By the combination of our assays and MALDI-TOF MS, we determined that the observations are most likely due to true enzymatic activity with sequence specificity cleavage. The use of the protease dimerization mutant provided a robust test of the role of protein concentration dictating the level of active enzyme. The work demonstrates product detection below the level of a single turnover. As such, this assay has utility even when concentrations of catalytically active dimer are low.

Though initial data suggest avenues for further development, this first iteration of the 3CL protease assay was not able to differentiate, with statistical significance, saliva samples derived from PCR-confirmed COVID-19-positive and -negative patients. Another work has recently demonstrated that the enzyme is detectable in COVID-19-positive saliva samples, adding support that our observations may truly be due to 3CL protease and are not just nonspecific [26]. This method has taken advantage of an antibody-mediated concentration of the enzyme occurring on the surface of a carbon paper strip. For future applications with clinical samples, a pre-concentration step will be employed to assess the diagnostic capabilities of multiplexed enzyme activity measurements. 

## Data Availability

Data are contained within the article and Supplementary Material.

## Supplementary Materials

The following supporting information can be downloaded at https://www.mdpi.com/article/10.3390/bios13070682/s1: Procedures for peptide synthesis. Figure S1. HPLC of 3CL protease substrate, Figure S2. Fluorescence standard curve of 3CL protease substrate, Figures S3–S5. Clinical samples data. Procedures for mutant protease production.

## References

[1] WHO Coronavirus (COVID-19) Dashboard. https://covid19.who.int.

[2] Wang H., Paulson K.R., Pease S.A., Watson S., Comfort H., Zheng P., Aravkin A.Y., Bisignano C., Barber R.M., Alam T. (2022). Estimating Excess Mortality Due to the COVID-19 Pandemic: A Systematic Analysis of COVID-19-Related Mortality, 2020–2021. Lancet.

[3] The Lancet Infectious Diseases (2022). Transitioning to Endemicity with COVID-19 Research. Lancet Infect. Dis..

[4] Weissleder R., Lee H., Ko J., Pittet M.J. (2020). COVID-19 Diagnostics in Context. Sci. Transl. Med..

[5] Au W.Y., Cheung P.P.H. (2021). Diagnostic Performances of Common Nucleic Acid Tests for SARS-CoV-2 in Hospitals and Clinics: A Systematic Review and Meta-Analysis. Lancet Microbe.

[6] Braunstein G.D., Schwartz L., Hymel P., Fielding J. (2021). False Positive Results With SARS-CoV-2 RT-PCR Tests and How to Evaluate a RT-PCR-Positive Test for the Possibility of a False Positive Result. J. Occup. Environ. Med..

[7] Healy B., Khan A., Metezai H., Blyth I., Asad H. (2021). The Impact of False Positive COVID-19 Results in an Area of Low Prevalence. Clin. Med..

[8] Liu C., Wang Z., Wu W., Xiang C., Wu L., Li J., Hou W., Sun H., Wang Y., Nie Z. (2021). Laboratory Testing Implications of Risk-Stratification and Management of COVID-19 Patients. Front. Med..

[9] Guo L., Ren L., Yang S., Xiao M., Chang D., Yang F., Dela Cruz C.S., Wang Y., Wu C., Xiao Y. (2020). Profiling Early Humoral Response to Diagnose Novel Coronavirus Disease (COVID-19). Clin. Infect. Dis..

[10] Ast V., Costina V., Eichner R., Bode A., Aida S., Gerhards C., Thiaucourt M., Dobler G., Geilenkeuser W.-J., Wölfel R. (2021). Assessing the Quality of Serological Testing in the COVID-19 Pandemic: Results of a European External Quality Assessment (EQA) Scheme for Anti-SARS-CoV-2 Antibody Detection. J. Clin. Microbiol..

[11] Zheng Q., Ma P., Wang M., Cheng Y., Zhou M., Ye L., Feng Z., Zhang C. (2022). Efficacy and Safety of Paxlovid for COVID-19:A Meta-Analysis. J. Infect..

[12] De Vito A., Colpani A., Bitti A., Zauli B., Meloni M.C., Fois M., Denti L., Bacciu S., Marcia C., Maida I. (2022). Safety and Efficacy of Molnupiravir in SARS-CoV-2-Infected Patients: A Real-Life Experience. J. Med. Virol..

[13] CDC Labs. https://www.cdc.gov/coronavirus/2019-ncov/lab/serology-testing.html.

[14] Peroni L.A., Toscaro J.M., Canateli C., Tonoli C.C.C., de Olivera R.R., Benedetti C.E., Coimbra L.D., Pereira A.B., Marques R.E., Proença-Modena J.L. (2021). Serological Testing for COVID-19, Immunological Surveillance, and Exploration of Protective Antibodies. Front. Immunol..

[15] Kwong G.A., Ghosh S., Gamboa L., Patriotis C., Srivastava S., Bhatia S.N. (2021). Synthetic Biomarkers: A Twenty-First Century Path to Early Cancer Detection. Nat. Rev. Cancer.

[16] Oliveira-Silva R., Sousa-Jerónimo M., Botequim D., Silva N.J.O., Paulo P.M.R., Prazeres D.M.F. (2020). Monitoring Proteolytic Activity in Real Time: A New World of Opportunities for Biosensors. Trends Biochem. Sci..

[17] Jiménez D., Martínez-Sanz J., Sainz T., Calvo C., Méndez-Echevarría A., Moreno E., Blázquez-Gamero D., Vizcarra P., Rodríguez M., Jenkins R. (2022). Differences in Saliva ACE2 Activity among Infected and Non-Infected Adult and Pediatric Population Exposed to SARS-CoV-2. J. Infect..

[18] Daniell H., Nair S.K., Esmaeili N., Wakade G., Shahid N., Ganesan P.K., Islam M.R., Shepley-McTaggart A., Feng S., Gary E.N. (2022). Debulking SARS-CoV-2 in Saliva Using Angiotensin Converting Enzyme 2 in Chewing Gum to Decrease Oral Virus Transmission and Infection. Mol. Ther..

[19] Hersh E.V., Wolff M., Moore P.A., Theken K.N., Daniell H. (2022). A Pair of “ACEs”. J. Dent. Res..

[20] Malard L., Kakinami L., O’Loughlin J., Roy-Gagnon M.-H., Labbe A., Pilote L., Hamet P., Tremblay J., Paradis G. (2013). The Association between the Angiotensin-Converting Enzyme-2 Gene and Blood Pressure in a Cohort Study of Adolescents. BMC Med. Genet..

[21] Zhao D., Cheng T., Koohi-Moghadam M., Wu M.-Z., Yu S.Y., Ding X., Pelekos G., Yiu K.H., Jin L. (2022). Salivary ACE2 and TMPRSS2 Link to Periodontal Status and Metabolic Parameters. Clin. Transl. Discov..

[22] Trougakos I.P., Stamatelopoulos K., Terpos E., Tsitsilonis O.E., Aivalioti E., Paraskevis D., Kastritis E., Pavlakis G.N., Dimopoulos M.A. (2021). Insights to SARS-CoV-2 Life Cycle, Pathophysiology, and Rationalized Treatments That Target COVID-19 Clinical Complications. J. Biomed. Sci..

[23] Jin Z., Mantri Y., Retout M., Cheng Y., Zhou J., Jorns A., Fajtova P., Yim W., Moore C., Xu M. (2022). A Charge-Switchable Zwitterionic Peptide for Rapid Detection of SARS-CoV-2 Main Protease. Angew. Chem. Int. Ed..

[24] Feng Y., Liu G., La M., Liu L. (2022). Colorimetric and Electrochemical Methods for the Detection of SARS-CoV-2 Main Protease by Peptide-Triggered Assembly of Gold Nanoparticles. Molecules.

[25] Garland G.D., Harvey R.F., Mulroney T.E., Monti M., Fuller S., Haigh R., Gerber P.P., Barer M.R., Matheson N.J., Willis A.E. (2022). Development of a Colorimetric Assay for the Detection of SARS-CoV-2 3CLpro Activity. Biochem. J..

[26] Borberg E., Granot E., Patolsky F. (2022). Ultrafast One-Minute Electronic Detection of SARS-CoV-2 Infection by 3CLpro Enzymatic Activity in Untreated Saliva Samples. Nat. Commun..

[27] Yan G., Li D., Lin Y., Fu Z., Qi H., Liu X., Zhang J., Si S., Chen Y. (2021). Development of a Simple and Miniaturized Sandwich-like Fluorescence Polarization Assay for Rapid Screening of SARS-CoV-2 Main Protease Inhibitors. Cell Biosci..

[28] Dražić T., Kühl N., Leuthold M.M., Behnam M.A.M., Klein C.D. (2021). Efficiency Improvements and Discovery of New Substrates for a SARS-CoV-2 Main Protease FRET Assay. SLAS DISCOVERY Adv. Sci. Drug Discov..

[29] Coelho C., Gallo G., Campos C.B., Hardy L., Würtele M. (2020). Biochemical Screening for SARS-CoV-2 Main Protease Inhibitors. PLoS ONE.

[30] Cao W., Cho C.-C.D., Geng Z.Z., Shaabani N., Ma X.R., Vatansever E.C., Alugubelli Y.R., Ma Y., Chaki S.P., Ellenburg W.H. (2022). Evaluation of SARS-CoV-2 Main Protease Inhibitors Using a Novel Cell-Based Assay. ACS Cent. Sci..

[31] Legare S., Heide F., Bailey-Elkin B.A., Stetefeld J. (2022). Improved SARS-CoV-2 Main Protease High-Throughput Screening Assay Using a 5-Carboxyfluorescein Substrate. J. Biol. Chem..

[32] Pijuan-Galito S., Tarantini F.S., Tomlin H., Jenkins H., Thompson J.L., Scales D., Stroud A., Tellechea Lopez A., Hassall J., McTernan P.G. (2021). Saliva for COVID-19 Testing: Simple but Useless or an Undervalued Resource?. Front. Virol..

[33] Santos L.H., Kronenberger T., Almeida R.G., Silva E.B., Rocha R.E.O., Oliveira J.C., Barreto L.V., Skinner D., Fajtová P., Giardini M.A. (2022). Structure-Based Identification of Naphthoquinones and Derivatives as Novel Inhibitors of Main Protease Mpro and Papain-like Protease PLpro of SARS-CoV-2. J. Chem. Inf. Model..

[34] Bartolowits M.D., Xin M., Petrov D.P., Tague T.J., Davisson V.J. (2019). Multimeric Rhodamine Dye-Induced Aggregation of Silver Nanoparticles for Surface-Enhanced Raman Scattering. ACS Omega.

[35] Suresh V., Byers K., Rajesh U.C., Caiazza F., Zhu G., Craik C.S., Kirkwood K., Davisson V.J., Sheik D.A. (2022). Translation of a Protease Turnover Assay for Clinical Discrimination of Mucinous Pancreatic Cysts. Diagnostics.

[36] Fink E.A., Bardine C., Gahbauer S., Singh I., White K., Gu S., Wan X., Ary B., Glenn I., O’Connell J. (2022). Large Library Docking for Novel SARS-CoV-2 Main Protease Non-Covalent and Covalent Inhibitors. bioRxiv.

[37] Tropea J.E., Cherry S., Waugh D.S. (2009). Expression and Purification of Soluble His(6)-Tagged TEV Protease. Methods Mol. Biol..

[38] Stamplecoskie K.G., Scaiano J.C., Tiwari V.S., Anis H. (2011). Optimal Size of Silver Nanoparticles for Surface-Enhanced Raman Spectroscopy. J. Phys. Chem. C.

[39] Keller B.O., Li L. (2006). Three-Layer Matrix/Sample Preparation Method for MALDI MS Analysis of Low Nanomolar Protein Samples. J. Am. Soc. Mass Spectrom..

[40] Sedlak S.M., Schendel L.C., Gaub H.E., Bernardi R.C. (2020). Streptavidin/Biotin: Tethering Geometry Defines Unbinding Mechanics. Sci. Adv..

[41] Söder P.-Ö., Modéer T. (1977). Characterization of Trypsin-like Enzymes from Human Saliva Isolated by Use of Affinity Chromatography. Acta Odontol. Scand..

[42] Rao G.J.S., Nadler H.L. (1972). Deficiency of Trypsin-like Activity in Saliva of Patients with Cystic Fibrosis. J. Pediatr..

[43] Hsu J.T.-A., Kuo C.-J., Hsieh H.-P., Wang Y.-C., Huang K.-K., Lin C.P.-C., Huang P.-F., Chen X., Liang P.-H. (2004). Evaluation of Metal-Conjugated Compounds as Inhibitors of 3CL Protease of SARS-CoV. FEBS Lett..

[44] Lee C.-C., Kuo C.-J., Hsu M.-F., Liang P.-H., Fang J.-M., Shie J.-J., Wang A.H.-J. (2007). Structural Basis of Mercury- and Zinc-Conjugated Complexes as SARS-CoV 3C-like Protease Inhibitors. FEBS Lett..

[45] Silvestrini L., Belhaj N., Comez L., Gerelli Y., Lauria A., Libera V., Mariani P., Marzullo P., Ortore M.G., Palumbo Piccionello A. (2021). The Dimer-Monomer Equilibrium of SARS-CoV-2 Main Protease Is Affected by Small Molecule Inhibitors. Sci. Rep..

[46] Nashed N.T., Aniana A., Ghirlando R., Chiliveri S.C., Louis J.M. (2022). Modulation of the Monomer-Dimer Equilibrium and Catalytic Activity of SARS-CoV-2 Main Protease by a Transition-State Analog Inhibitor. Commun. Biol..

[47] Zhang L., Lin D., Sun X., Curth U., Drosten C., Sauerhering L., Becker S., Rox K., Hilgenfeld R. (2020). Crystal Structure of SARS-CoV-2 Main Protease Provides a Basis for Design of Improved α-Ketoamide Inhibitors. Science.

