# Supplementary material for: A Prototype Assay Multiplexing SARS-CoV-2 3CL-Protease and Angiotensin-Converting Enzyme 2 for Saliva-Based Diagnostics in COVID-19
Source: Biosensors (Basel). 2023 Jun 27;13(7):682. doi: 10.3390/bios13070682 (PMC10377347; doi:10.3390/bios13070682)
Supplement: Supplementary file 1 [file biosensors-13-00682-s001.zip › biosensors-2351279-supplementary.pdf]

SUPPORTING MATERIALS

PEPTIDE SYNTHESIS 3CL peptide substrate was synthesized using a CEM Liberty Blue Microwave Peptide Synthesizer on Octagel resin (0.05 mmol; 0.32 mmol/g, AAPTEC), washed 3x and pre-swelled with 5 mL of DMF. All equivalences are with respect to resin loading. Couplings were performed at 90 ^{o}C with 2 eq. of Fmoc- amino acids, 2 eq. of DIC, and 2 eq. of Oxyma in DMF. Deprotections were performed using 20% piperidine in DMF. Between each step, the resin was washed with 5 mL DMF. After the final amino acid deprotection, dimeric rhodamine dye (1.1 eq.) was coupled using 1.1 eq. HCTU and 3 eq. DIEA for 24 hours at room temperature. The resin was then washed 3x each with DMF, MeOH, and DCM. Cleavage from resin was performed using trifluoracetic acid, TIPS, and water (95:2.5:2.5) for 2 hours at room temperature. The solvent was removed in vacuo and the crude peptide precipitated using cold diethyl ether. The solids were isolated by centrifugation and dissolved in water for purification by HPLC.

For semi-preparative separations, a Grace Altima C18 column was used. The compound was purified by eluting with water and acetonitrile, each containing 0.01% trifluoroacetic acid (solvent A and solvent B respectively). A gradient elution, with a constant flow rate of 3 mL/min., was performed: 5% solvent B in A for the first 5 minutes, followed by a linear increase from 5% solvent B to 95% solvent B from until 20 min., a wash period of 95% solvent B from 20 to 25 min., and finally a linear decrease from 95% to 5% solvent B in A from 25 to 30 min. The desired product (eluting at 18.4 minutes, strong absorption at 216 nm and 520 nm) was collected as fractions, combined, and dried to give 16 mg of pure product (11.7% overall yield). The product was confirmed by matrix assisted laser desorption/ionization mass spectrometry, performed using a Voyager DE Spectrometer (M+: Calc: 2823.58, Found: 2823.71).

For purity analysis, a Vydac Protein and Peptides C18 column was used. A gradient elution, with a constant flow rate of 1 mL, was performed: 5% solvent B in A for the first 5 minutes, followed by a linear increase from 5% solvent B to 95% solvent B from until 20 min., a wash period of 95% solvent B from 20 to 25 min., and finally a linear decrease from 95% to 5% solvent B in A from 25 to 30 min. The compound was eluted at 17.34 minutes. Analysis of peak areas determined that the peptide was >99% pure (**Fig. S1**).

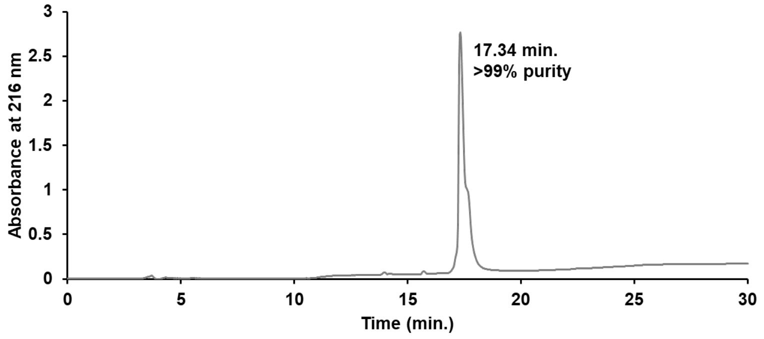

Figure S1. HPLC of 3CL protease substrate

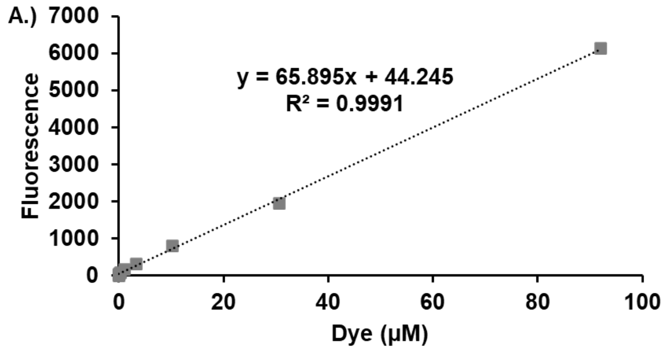

Figure S2. Standard curve of 3CL substrate dye to normalize y-axes for other plots.

CLINICAL SAMPLES HANDLING Saliva samples were collected at Neelyx Labs (Wood Dale, IL, USA) under an IRB approval (HIRB Project No. 092222-416, Approved September 22, 2022), frozen at -20°C for storage, and thawed prior to running assays. Assays were run according to the sequential procedure described previously. Fluids generated in these assays were then deactivated by heating, transported to Amplified Sciences Inc. (West Lafayette, IN, USA), and read on a Biotek plate reader. Fluorescence values from each assay were plotted in bar graphs and box-and-whisker plots for analysis. To compare groups, a 2 tailed paired t-test was used.

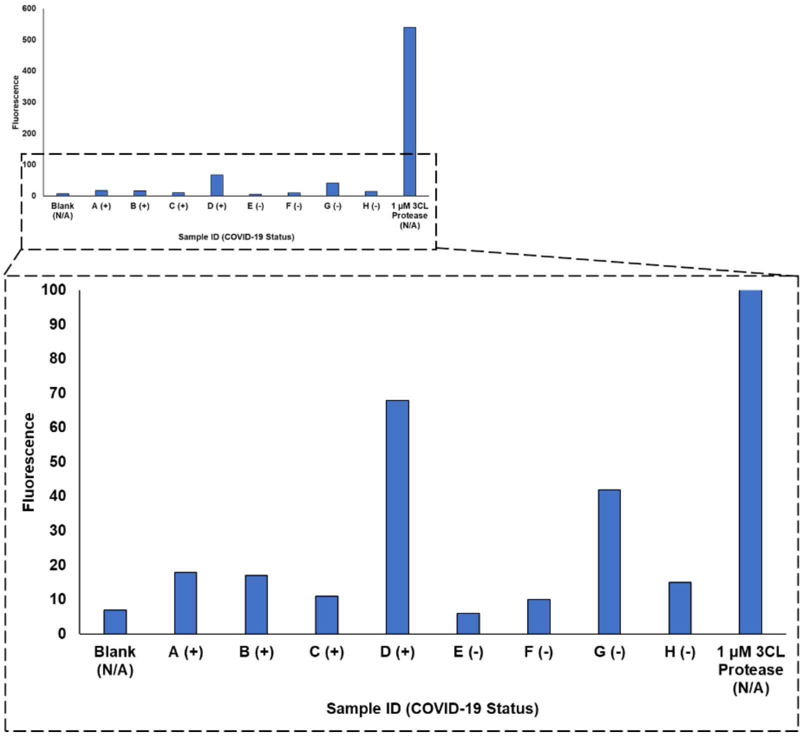

Figure S3. 20 minute procedure 3CL protease assays in clinical samples (A-H), along with a negative (Blank) and positive control (1 µM 3CL Protease)

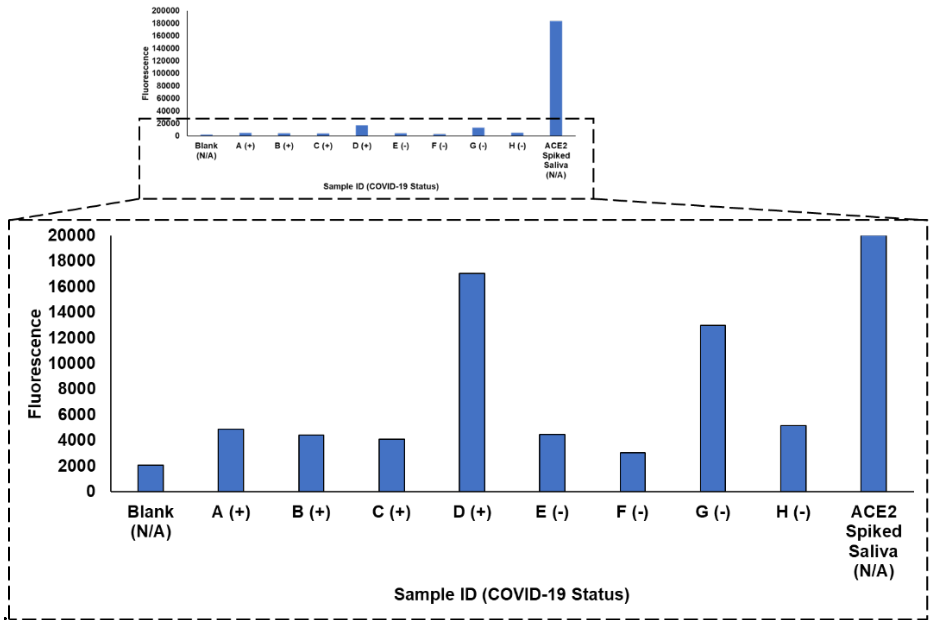

Figure S4. Duplex procedure ACE2 assays in clinical samples (A-H), along with a negative (Blank) and positive control (ACE2 spiked saliva)

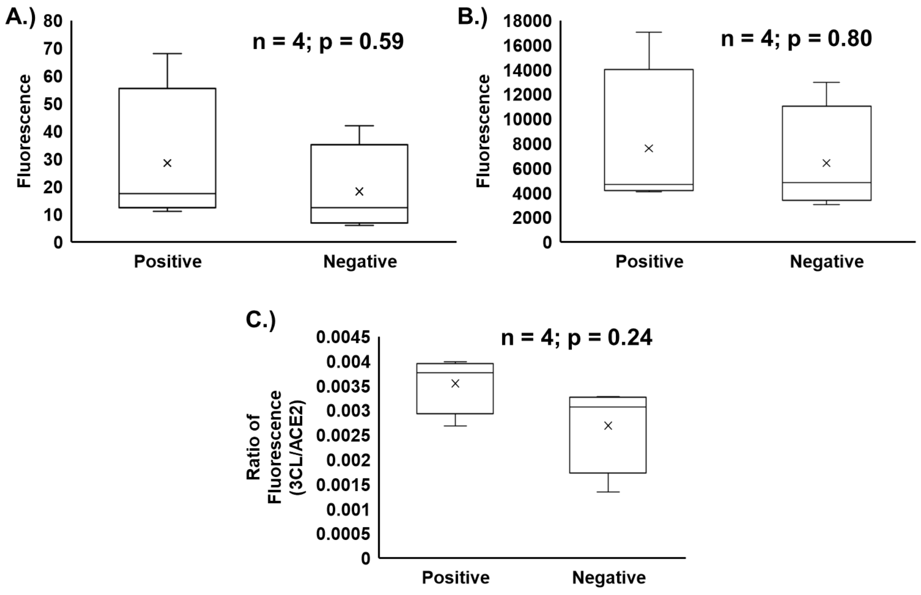

Figure S5. A.) There is no statistically significant difference in 3CL-protease-generated fluorescence between COVID-19 positive and negative samples; the former trends higher very weakly. B.) There is no statistically significant difference in ACE2-generated fluorescence between COVID-19 positive and negative samples; the former trends higher very weakly. C.) The ratio between 3CL protease and ACE2 assay signals differentiates the samples better than the individual values but even this does not reach statistical significance.

PREPARATION OF MONOMERIC 3CL PROTEASE MUTANT A construct for bacterial expression modified from a previous report as used.[1] This construct contains an N-terminal GST domain to facilitate soluble expression followed by the native cleavage sequence and the 3CL protease with a HRV 3C cleavable polyhistidine tag. To obtain the monomeric variant of the protein, this construct was modified to contain an extra N-terminal SN by altering the native cleavage site to a TEV cleavable sequence specifically engineered to leave the extra residues. The Q5 site-directed mutagenesis kit (NEB) was used with the following primers per the manufacturer's protocol. BL21 DE3 Rosetta 2 PLysS bacteria were transformed with the resulting construct. A single colony was first grown overnight at 37 ° C in 2XYT supplemented with 2% Glucose, 100 μg/mL ampicillin and 20 μg/mL chloramphenicol. Fresh 2XYT media supplemented with 2% Glucose 100 μg/mL ampicillin and 20 μg/mL chloramphenicol was then inoculated with a 1:200 dilution of the overnight culture and allowed to reach 0.8 OD_{600}. The expression was then induced with 1 mM IPTG for 4 h at 37 ° C. The cells were harvested, pelleted through centrifugation at 4,000 x g for 20 min, and then frozen at -80 ° C before use. Frozen pellets were resuspended in Buffer A (20 mM HEPES, 10 mM Imidazole, 400 mM NaCl pH 8.0) with 0.1 mg/mL DNAse I (Sigma-Aldrich, St. Louis, MO) and disrupted through sonication. The lysate was then clarified through centrifugation at 16,000 x g for 45 min. The clarified lysate was then gently passed over a pre-equilibrated (Buffer A) column containing Ni-IMAC His60 resin (Takara Bio, San Jose, CA). The column was washed with 20 CV of Buffer A, and then eluted through a step-gradient of increasing imidazole in Buffer A (40 mM, 100 mM, 250 mM, and 500 mM imidazole). Fractions were analyzed by SDS-PAGE and fractions containing the construct were pooled and concentrated using 10 KDa MWCO centrifugal filters (Millipore, Burlington, MA) to a 1 mL volume. Protein concentration was determined by A280 and his tagged HRV3C was added (1mg HRV3C: 50 mg of protein) (ThermoFisher Scientific, Waltham, MA) and dialyzed using Slide-A-Lyzer 10k MWCO cassette (ThermoFisher Scientific, Waltham, MA) O/N at RT in Buffer B (50 mM Tris 150 mM NaCl 5 mM DTT pH 7.5) to remove the hexahistidine tag. The reaction was then passed over a pre-equilibrated Ni-IMAC column with Buffer A to remove the HRV3C protease and any noncleaved protein. Protein concentration was determined by A280, and hexahistidine tagged TEV protease that was prepared and added to the pooled protein.[2] This mixture was then dialyzed at RT for 8 h against Buffer B) with Slide-A-Lyzer 10k MWCO cassette. The contents of the dialysis cassette were then passed over pre-equilibrated Ni-NTA resin (Buffer A) to remove the tagged TEV protease. The resin was washed with Buffer A and pooled with the flowthrough and passed over a pre-equilibrated (25 mM HEPES 150 mM NaCl pH 7.5) Pierce™ Glutathione Agarose GSH-resin (ThermoFisher Scientific Waltham, MA) to remove the cleaved GST domain and then eluted with 10 mM GSH 25 mM HEPES 150 mM NaCl pH 7.5. The protein was analyzed by SDS-PAGE and then concentrated using 10 KDa MWCO centrifugal filters. and buffer exchanged by passing over a PD-10 column pre-equilibrated with Buffer C (25 mM HEPES 1 mM TCEP 150 mM NaCl pH 7.5) the protein was concentrated, aliquoted, and flash frozen using liquid N_2. The protein was then stored at -80 ° C before use in assays.

1. Fink, E.A.; Bardine, C.; Gahbauer, S.; Singh, I.; White, K.; Gu, S.; Wan, X.; Ary, B.; Glenn, I.; O'Connell, J.; et al. Large Library Docking for Novel SARS-CoV-2 Main Protease Non-Covalent and Covalent Inhibitors 2022, 2022.07.05.498881.
2. Tropea, J.E.; Cherry, S.; Waugh, D.S. Expression and Purification of Soluble His(6)-Tagged TEV Protease. *Methods Mol Biol* **2009**, *498*, 297–307, doi:10.1007/978-1-59745-196-3_19.
